# Topical Steroid Damaged Face: A Cross-Sectional Study from Saudi Arabia

**DOI:** 10.3390/clinpract12010018

**Published:** 2022-02-21

**Authors:** Mahdi Al Dhafiri, Alaa Baqer Alali, Zuhur Ali Alghanem, Zahraa Wasel Alsaleh, Eman Abdulrahman Boushel, Zahraa Baqer Alali, Aeshah Adel Alnajjar

**Affiliations:** 1 Department of Dermatology, College of Medicine, King Faisal University, Al-Ahsa 31982, Eastern Province, Saudi Arabia; 2 College of Medicine, King Faisal University, Al-Ahsa 31982, Eastern Province, Saudi Arabia; alaaalali928@gmail.com (A.B.A.); zuhuralghanem@gmail.com (Z.A.A.); zahraawasel17@gmail.com (Z.W.A.); eman-5006@hotmail.com (E.A.B.); zhrzhr13579@gmail.com (Z.B.A.); aishadel1999@gmail.com (A.A.A.)

**Keywords:** topical corticosteroid, face, side effects, topical steroid damaged face

## Abstract

Corticosteroids are one of the anti-inflammatory drugs that are used widely by dermatologists. Significant local adverse effects can happen if topical corticosteroids (TCs) are used incorrectly. This study aimed to assess the prevalence of facial TCs misuse and its adverse effects. This was cross-sectional research: a self-reported questionnaire was distributed among a population of Saudi Arabians aged 16 years and above who were using TCs consecutively. Statistical analysis was performed using SPSS version 26. A total of 611 participants were enrolled in the survey: 401 (65.6%) were university graduates, while 187 (30.6%) were below high school level of education. The number of participants using TCs was 279 (45.7%), while 332 (54.3%) did not use steroids topically. The most used TCs were Mometasone furoate 0.1% cream (18.2%), followed by Fusidic acid/Betamethasone cream (16.7%). A total of 46 reported facial TCs’ side effects. Peeling (52.2%) was the most reported side effect, followed by redness (41.3%). In conclusion, the use of facial TCs among the Saudi population is not uncommon (16.5%). A large population are not aware of the side effects of the unsupervised use of TCs. An effort should be made to increase awareness of the adverse effects of TCs.

## 1. Introduction

In 1950, Philip Hench, Edward Kendall, and Tadeus Reichstein discovered the corticosteroid hormone [1]. Corticosteroids are a type of anti-inflammatory drug used in many specialties of medicine [2].

Topical corticosteroid is the synthetic form of natural corticosteroid which is synthesized in the adrenal cortex [3]. Topical corticosteroids are widely used by dermatologists and are highly effective in treating a variety of dermatological disorders. Topical corticosteroids (TCs) have a rapid effect in controlling dermatologic-inflammatory conditions due to their anti-inflammatory, anti-pruritic, and immunosuppressive effects on the skin [4]. There are different potencies of TCs ranging from mild to very potent. They are used over different body parts, including the face. Different dermatologic conditions are treated with TCs, including atopic dermatitis and psoriasis [5]. However, the side effects of steroids are unneglectable; they are ranging from mild to very severe depending on the area exposed to the TCs, period, and frequency of usage. Side effects are notable on soft, sensitive areas of the body with a high rate of transcutaneous absorption, such as the face. These adverse effects can include acne, telangiectasia, steroids rosacea, and hyper/hypopigmentation. The risk of these adverse effects increases with the long-term use of TCs [6]. Topical steroid damaged face (TSDF) was a newly described phenomenon in 2008, which is characterized by a group of symptoms induced by the prolonged, unsupervised usage of TCs on the face, regardless of the potency [6]. In this study, we aimed to assess the prevalence of TSDF among the population of Saudi Arabia and the associated adverse effects.

## 2. Materials and Methods

A community-based, cross-sectional study was conducted. A validated self-report questionnaire was distributed among participants to assess the adverse side effects of using facial TCs. The inclusion criteria were: (1) aged 16 years or above; (2) from Saudi Arabia; (3) male or female. The study proposal was ethically approved by the ethical committee of the scientific research committee at King Fahad Hofuf Hospital. Data collection was conducted by an online questionnaire using Google Forms. The questionnaire was validated after being designed by two expert consultant dermatologists. The next step included a pilot study through distributing the questionnaire to over 25 participants. Then, this process was followed by cleaning the collected data to reduce the risk of error, using principal components analysis, checking the internal consistency, and revising the survey as a final stage. The validated questionnaire was distributed by social media to the Saudi Arabian population. We had volunteers from each region who ensured that the questionnaire was delivered equally to all Saudi regions. The participants had given their consent to participate in the study before filling out the questionnaire. The questionnaire included the demographic data of the participants (age, gender, nationality, place of residence, marital status, education, and employment status). Then, it was followed by questions about the usage of TCs.

The population aged 16 and above who live in Saudi Arabia are 25,828,206, depending on the data available in the General Authority for Statistics of Saudi Arabia [7]. The sample size was calculated using this formula by *n* = z2pq\d2. The confidence level was 95%, the estimated proportion was 50%, with a 5% level of precision. The minimum sample size was calculated to be 385. However, more participants were included to ensure accuracy.

Data analysis was performed after data were extracted. It was revised, coded, and fed to statistical software IBM SPSS version 22 (SPSS, Inc. Chicago, IL, USA). All statistical analysis was conducted using two-tailed tests. A *p*-value less than 0.05 was statistically significant. Descriptive analysis based on frequency and percent distribution was performed for all variables, including participants’ socio-demographic data, facial TCs use, types of used steroids, frequency of use, causes of use, TCs complications, and their satisfaction regarding used topical facial steroids. Cross tabulation was used to assess the distribution of facial TCs use according to their socio-demographic data. Relations were tested using Pearson’s chi-square test and exact probability test for small frequency distributions.

## 3. Results

A total of 611 participants fulfilling the inclusion criteria completed the study questionnaire. The participants ranged from 16 to 67 years, with a mean age of 29.1 ± 11.6 years old. An exact number of 494 (80.9%) participants were female. As for educational level, 401 (65.6%) were university graduates, 187 (30.6%) were high school graduates, and (22.3%) were below high school. A total of 267 (43.7%) respondents were students, 136 (22.3%) had governmental jobs, and 160 (26.2%) were not employed. Among participants, 62 (13.7%) were physicians, 22 (4.9%) were nurses, and 9 (2%) were pharmacists. As for the prevalence of using facial steroids, 279 (45.7%) study participants used TCs, while 332 (54.3%) did not use TCs.

The most used type of TCs were Mometasone furoate 0.1% cream (18.2%), followed by Fusidic acid/betamethasone cream (16.7%), Hydrocortisone 1% cream (4.4%), and Betamethasone valerate 0.1% cream (0.8%). A total of 41.6% of the facial topical steroids users applied it to cheeks, while 21.5% used TCs on the forehead, 21.1% applied TCs around the mouth, 3.9% around their eyes, while 30.5% applied TCs for the whole face. Regarding the causes of using TCs, 45.9% of the users used for acne, followed by 30.5% for eczema/dermatitis, 14% used TCs to lighten their skin, 5% for freckles, and 5% for psoriasis. Regarding the frequency of using TCs, 35.1% applied TCs once daily, 16.1% applied steroids twice daily, and 7.2% applied every 2 weeks. Exactly 44.1% of TCs users had the cream form, while 52% had ointment, and 3.9% used other forms. Using TCs for 1–2 months was reported among 38% of the users, while 42.3% used topical steroids for more than 9 months. An exact number of 102 (36.6%) participants who used the TCs reported still using it (Table 1).

Nevertheless, 154 (55.2%) users had facial TCs based on a doctor’s prescription. Among those who used unprescribed TCs, family & friends were the main source of advice to use (41.6%), followed by pharmacists (25.6%), social media (18.4%), and books (5.6%). Only 77 (27.6%) TCs users followed up with a dermatologist to note the effect of using TCs on the face. Side effects due to the use of facial TCs were reported among 46 (16.5%) participants. The most reported side effects were peeling (52.2%), followed by redness (41.3%), dryness (23.9%), skin color change (21.7%), irritation (19.6%), acne-like rash (10.9%), and itching (10.9%). A burning sensation was one of the least reported side effects (6.5%), along with skin stretch marks (2.2%). An exact number of 108 (38.7%) knew the side effects of using TCs, and 23 (50%) of those with side effects reported they would return to use TCs if the side effects improved (Table 2).

A total of 93 (33.3%) users were not satisfied, 78 (28%) were moderately satisfied, and 108 (38.7%) TCs users were highly satisfied. A total of 127 (68.3%) steroid users needed less than 1 month to be satisfied, while 39 (21%) needed 1–3 months, but 5 (2.7%) needed more than 9 months. A total of 167 (59.9%) participants did not use anything after they stopped using TCs, while 53 (19%) used another type of cream, and 50 (17.9%) used other methods. As for participants’ observations on their skin after stopping TCs use, 161 (65.7%) observed no change in the treatment result, 65 (26.5%) still had the same problem as before using cortisone, and 12 (4.9%) had persistent side effects (Table 3).

A total of 54.5% of participants aged less than 20 years used TCs, compared to 30.5% of those aged 50 years or more with recorded statistical significance (*p* = 0.001). Moreover, TCs use was recorded among 47.8% of females, in comparison with 36.8% of males (*p* = 0.031). A total of 51.7% of the students used facial TCs versus 35.4% of workers at non-governmental settings (*p* = 0.029). Furthermore, facial TCs use was reported among 77.3% of nurses, compared to 43.5% of physicians (*p* = 0.027) (Table 4).

## 4. Discussion

Corticosteroids are a type of anti-inflammatory drug that can be prescribed in a systemic or topical form. In 1951, topical corticosteroids (TCs) were used for the first time by American dermatologist Marion Baldur Sulzberger [2]. In modern dermatological practice, TCs have become one of the most widely used treatment methods [3].

Regarding the prevalence of using facial TCs among the Saudi population, this study shows that 279 (45%) participants used facial topical steroids, while 332 (54%) did not use facial topical steroids. Topical steroids usage is more popular among females than the male population, which is similar to what was reported in previous studies [6,8]. Most of our participants were university graduates or below high school education level. The most used TCs in this study was Mometasone furoate 0.1% cream (18.2%), followed by Fusidic acid/betamethasone cream (16.7%), Hydrocortisone 1% cream (4.4%), and Betamethasone valerate 0.1% cream (0.8%). This is similar to the result: mid potent formulation (mometasone) was the most common overused TCs [6]. This can be explained by the ever-increasing popularity of triple combinations (mometasone, hydroquinone, and tretinoin) for fairness creams [6].

The most common reason for participants to use facial TCs in this study was treating acne (45.9%). This can be explained by the fact that acne vulgaris is one of the most common inflammatory disorders, affecting a large percentage of the young population in Saudi Arabia (78%) [9]. In a similar study conducted in India, the most common indication of topical steroids was skin lightening (70.6%), followed by treating acne (12.9%) [6]. Locally, the study conducted in Hail, Saudi Arabia, had shown that the most common reason for using topical steroids was skin lightening [3].

In this study, the most commonly used form of TCs was ointment in a frequency of once daily for more than nine months, while the average duration in a previous, similar study was around 6 ± 2 months [6]. However, in another study conducted in Hail, the majority of participants (67%) used TCs for less than three months [10].

This study exhibits that 55.2% of the users had facial topical steroids based on doctor prescriptions. This matched the results of another study: 44% of women who used the topical steroids received the prescription from a doctor [10]. Among those who used unprescribed topical steroids, family and friends were the main sources of advice, similar to the other local study, which revealed that the leading inspirations toward the usage of facial TCs were relatives and friends [3,5].

Because the nature of facial skin is thinner than other parts of the body, with a high transcutaneous absorption rate, side effects are more common on the face [3]. Nevertheless, only 16.5% of the participants of this study report any side effects of using TCs. This could be related to prescription restrictions in the kingdom, which participates in limiting TCs misuse. However, our study reveals some side effects, including peeling (52.2%), redness, dryness, skin color change, irritation, acne-like rash, and itching. New-onset acne or exacerbation of preexisting acne represented the single most important adverse event. This was followed by erythema/telangiectasia [6]. Among our participants, 61.3% were unaware of the side effects of using TCs, while in the Indian study, 83.76% were unaware of the side effects of the TCs [5]. Exactly 50% of those with side effects reported their willingness to reuse cortisone if the side effects improved.

Regarding the satisfaction of TCs use, 38.7% were highly satisfied. Most patients (68.3%) needed less than one month to be satisfied. The majority of our participants (59.9%) did not use anything after they stopped using TCs. However, only a few (3.2%) went to a dermatologist for side effects. The observations on the skin after stopping TCs, 65.7% observed no change in the treatment result.

## 5. Conclusions

The usage of facial TCs among the Saudi population is not uncommon. Topical facial steroids side effects are not very common in Saudi Arabia (16.5%). A large population are not aware of the side effects of unsupervised usage of TCs. Efforts should be made to increase awareness about the adverse effects of topical TCs.

## Figures and Tables

**Table 1:** Distribution of topical corticosteroids use among participants.

Topical Steroids Use Data	No (279)	%
Used topical cortisone		
None	332	54.3%
Mometasone	111	18.2%
Fusidic acid/betamethasone	102	16.7%
Others	34	5.6%
Hydrocortisone	27	4.4%
Betamethasone	5	0.8%
In which part of the face is topical cortisone used?		
Cheeks	116	41.6%
All face	85	30.5%
Forehead	60	21.5%
Others	60	21.5%
Peri-oral	59	21.1%
Around the eyes	11	3.9%
Causes of using local CS		
Acne	128	45.9%
Eczema & dermatitis	85	30.5%
Others	73	26.2%
To lighten the skin	39	14.0%
Freckles	14	5.0%
Psoriasis	14	5.0%
How often do you apply topical cortisone?		
2 times/daily	45	16.1%
1 time/daily	98	35.1%
Every 2 days	13	4.7%
Every 3 days	13	4.7%
Every week	9	3.2%
Every 2 weeks	20	7.2%
Others	81	29.0%
Type of topical cortisone used		
Cream	123	44.1%
Ointment	145	52.0%
Others	11	3.9%
How long have you used topical cortisone on the face?		
1–2 months	106	38.0%
2–3 months	29	10.4%
3–6 months	18	6.5%
6–9 months	8	2.9%
>9 months	118	42.3%
Still using topical cortisone on the face?		
Yes	102	36.6%
No	177	63.4%

**Table 2:** Distribution of topical corticosteroids prescribed use and its complications participants.

Topical Steroid Prescription & Complications	No (279)	%
Used topical cortisone on the face based on a doctor’s prescription?		
Yes	154	55.2%
No	125	44.8%
If no, what is the source of your information? (*n* = 125)		
Family/friends	52	41.6%
Pharmacist	32	25.6%
Social media	23	18.4%
Books	7	5.6%
Others	11	8.8%
Follow up with a dermatologist to note the effect of using topical cortisone on the face?		
Yes	77	27.6%
No	202	72.4%
Had any side effects from using topical cortisone?		
Yes	46	16.5%
No	233	83.5%
If yes, what were these side effects? (*n* = 46)		
Peeling	24	52.2%
Redness	19	41.3%
Dryness	11	23.9%
Skin color change	10	21.7%
Irritation	9	19.6%
Acne-like rash	5	10.9%
Itching	5	10.9%
Burning sensation	3	6.5%
Skin stretch marks	1	2.2%
Did you know the side effects of using topical corticosteroids?		
Yes	108	38.7%
No	171	61.3%
Will you return to using cortisone if your side effects improve? (*n* = 46)		
Yes	23	50.0%
No	23	50.0%

**Table 3:** Distribution of participants’ satisfaction regarding topical steroid use.

Satisfaction Regarding Topical Steroid Use	No (279)	%
How satisfied are you with the results of topical cortisone on the face?		
Not satisfied (1–4)	93	33.3%
Moderate satisfaction (5–7)	78	28.0%
High satisfaction (8–10)	108	38.7%
Duration until a high satisfaction level		
<1 month	127	68.3%
1–3 months	39	21.0%
3–6 months	7	3.8%
6–9 months	8	4.3%
>9 months	5	2.7%
If you have stopped using topical corticosteroids on the face, what have you done after stopping?		
Used another type of cream	53	19.0%
I went to a dermatologist for side effects	9	3.2%
Others	50	17.9%
I did not use anything	167	59.9%
Observations on your skin after stopping topical cortisone use?		
Persistent side effects	12	4.9%
Worsening side effects	7	2.9%
Still have the problem present before using cortisone	65	26.5%
No change in the treatment result	161	65.7%

**Table 4:** Distribution of participants use of topical facial corticosteroids by their personal data.

Personal Data	Total No (%)	Use of Topical Corticosteroids on Face				*p*-Value
		Yes		No		
		No	%	No	%	
Age in years						0.001
<20	112 (18.3%)	61	54.5%	51	45.5%	
20–29	282 (46.2%)	144	51.1%	138	48.9%	
30–49	158 (25.9%)	56	35.4%	102	64.6%	
50+	59 (9.7%)	18	30.5%	41	69.5%	
Gender						0.031
Male	117 (19.1%)	43	36.8%	74	63.2%	
Female	494 (80.9%)	236	47.8%	258	52.2%	
Educational level						0.115
Below high school	23 (3.8%)	9	39.1%	14	60.9%	
High school	187 (30.6%)	97	51.9%	90	48.1%	
University/above	401 (65.6%)	173	43.1%	228	56.9%	
Are you a health care worker?						0.027
Physician	62 (13.7%)	27	43.5%	35	56.5%	
Pharmacist	9 (2.0%)	4	44.4%	5	55.6%	
Nurse	22 (4.9%)	17	77.3%	5	22.7%	
No	358 (79.4%)	159	44.4%	199	55.6%	

## Data Availability

All data associated with this study are available and will be provided upon editor’s request.
